# Charting bioethical frontiers: China’s human organoid guidelines in a global context

**DOI:** 10.1186/s40779-025-00651-x

**Published:** 2025-10-09

**Authors:** Liang-Bin Zhou, Yi-Ting Lei, Xin-Xin Han

**Affiliations:** 1 Department of Biomedical Engineering, Faculty of Engineering, the Chinese University of Hong Kong, Hong Kong SAR, China; 2 InnoHK Center for Neuromusculoskeletal Restorative Medicine, Hong Kong Science and Technology Park, Hong Kong SAR, China; 3 Department of Orthopaedics, the First Affiliated Hospital of Chongqing Medical University & Orthopaedic Laboratory of Chongqing Medical University, Chongqing, 400016 China; 4 Organ Regeneration X Lab, Lisheng East China Institute of Biotechnology, Peking University, Nantong, 226299 Jiangsu China; 5 Shanghai Lisheng Biotech, Shanghai, 200092 China

**Keywords:** Human organoids, Regulatory policy, Ethical guidelines, Brain organoids, Embryo models, Organoid chimeras

The rapid progress of human organoid toolkits presents unprecedented opportunities in disease modeling, drug discovery, and personalized therapy, alongside profound bioethical challenges and concerns. On April 29th, 2025, China’s National Science and Technology Ethics Committee (Life Science Ethics Subcommittee) issued the Human Organoid Research Ethical Guidelines [1], establishing the world’s first comprehensive governance framework, especially focusing on brain organoids, embryo models, and chimeric research. This Guidelines represent a pioneering governance model in emerging biotechnology. This commentary offers an in-depth analysis to examine the policy’s innovative three-tiered structure, contrast it with current global regulatory standards, evaluate its domestic impacts as well as potential implications for international biotechnology governance, and provide recommendations for future directions.

Human organoids are three-dimensional multicellular miniature structures derived from the self-assembly of stem cells under specific culture conditions or from tissue explants such as tumor biopsies [2, 3]. The U.S. Food and Drug Administration (FDA) and European Commission have recently enacted policies actively promoting the integration of New Alternative Methodologies (NAMs), particularly human organoids, into the safety and efficacy assessments in preclinical studies while outlining commitments to phase out mandatory animal testing requirements [4–6]. This strategic shift is driven by the unparalleled advantages of organoids, which provide human-specific (patho)physiologically relevant data with superior predictive power, greater scalability, and enhanced ethical sustainability than traditional animal models [2, 3, 6]. Because human organoids can model intricate aspects of human biology (e.g., neural networks and early development) in ways that existing regulations cannot address, new bioethical frameworks are vital to manage associated risks of consciousness or cognition, blurred species boundaries (e.g., especially in nervous and reproductive systems), and early embryonic development. Brain organoids exhibiting neural oscillations, integrated stem cell-based embryo models (ISEMs) simulating early development, and organoid-chimeras forming cross-species biohybrids intensify debates on consciousness potential, synthetic embryogenesis, and risks of species integrity, human germline contamination, human neural integration, and consciousness hybridization, respectively [7–9]. While the International Society for Stem Cell Research (ISSCR) released general guideline in 2021 [7], no national regulatory authority had previously codified binding standards for collectively targeting brain organoids, organoid-chimeras, and ISEMs. Although clearer quantitative thresholds are still needed for effective implementation, China’s Guidelines profoundly bridge these gaps through a legally enforceable framework, signaling a shift from reactive to preemptive bioethics governance [1].

## Structural innovation of the guidelines: a tiered governance architecture

### Foundational principles: Confucian-ethical synthesis

The Guidelines anchor human organoid research into 5 core ethical principles that uniquely integrate Western bioethics with Eastern Confucian values [1] (Additional file 1: Fig. 1a). Beneficence prioritizes societal welfare over individual gains, reflecting Confucian communitarian norms. Risk control extends beyond human subjects to adjacent environmental protection, emphasizing holistic responsibility. Respect for autonomy adopts dynamic consent protocols but omits Western-style profit-sharing mandates. Scientific necessity aligns with resource efficiency traditions, demanding minimal biological material use. Fairness explicitly combats technology-driven stigmatization and discrimination, echoing socialist goals of equity and inclusiveness. The above foundational principles evidently diverge from ISSCR’s individual-centric framework by consistently elevating collective welfare, deliberately embedding China’s socio-ethical priorities into emerging biotech governance.

To operationalize these principles into practical decision-making, Research Ethics Committees (RECs) in China should adopt novel deliberative mechanisms that reflect this communitarian ethos. For instance, consider a proposal to create brain organoids from patients with a rare neurological disorder to study a neurotropic virus causing a regional epidemic. While a REC guided by ISSCR principles might prioritize individual donor autonomy and explicit profit-sharing and intellectual property agreements, a Chinese REC would likely employ a broader ethical calculus. Guided by communitarian ‘Beneficence’, the committee might place greater weight on the brain organoid research’s potential to alleviate community-wide crisis, potentially approving it with expedited review based on societal need. Furthermore, to embody ‘Fairness’ and prevent stigmatization, the RECs might mandate broader, community-level consultation even if individual consent protocols are less conventional. The community consultation process involving local patient advocacy groups and public health officials can better gauge societal concerns and ensure the research aligns with the public good rather than merely commercial interests. This case illustrates how abstract principles translate into practice. By formally weighing communal benefits alongside individual rights and introducing steps for collective dialogue, the RECs embeds China’s socio-ethical priorities directly into its decision-making architecture.

### Eight general requirements: operationalizing compliance

The Guidelines introduce several operational innovations among 8 general requirements [1] (Additional file 1: Fig. 1b). When conducting human organoid research, specialized RECs must include domain experts with relevant expertise (e.g., neurobiologists for brain organoids and developmental biologists for ISEMs), moving beyond generic Institutional Review Boards. Researchers must properly preserve and strictly manage all human genetic resources, biological materials, derived resources, and research results obtained during organoid studies. This systematic management, covering storage, sharing, and disposal, must comply with national laws, regulations, and ethical norms, ensuring traceability, safety, scientific integrity, and ethical compliance. Mandatory personnel certification of organoid research requires state-accredited training in specialized technical skills, laws, ethics, and safety. Crucially, dynamic consent protocols demand reconsent for research scope changes, while neural data classification treats electrophysiological outputs as sensitive health information, protecting the privacy of data donors. These mechanisms shift ethics from passive review to enforceable practice.

Specifically, the Guidelines’ mandate for the dynamic consent model represents a significant operational shift from single-point, blanket consent to a process of ongoing engagement and informed decision-making [1]. To illustrate its practical implementation, consider a hypothetical multi-phase study creating brain organoids from a donor with a genetic form of Parkinson’s disease. The initial consent would cover the derivation of induced pluripotent stem cells (iPSCs) and their applications for in vitro disease modeling. However, the dynamic consent protocol would activate tiered, opt-in checkpoints at each subsequent research phase. For example, before proceeding with electrophysiological recording to study neural network activity, which is classified as sensitive health data, donors would be recontacted to obtain authorization for this specific use. A further explicit reconsent would be required if researchers proposed a new aim, such as using clustered regularly interspaced short palindromic repeats (CRISPR)/CRISPR-associated protein 9 (Cas9) to introduce a different mutation. The most stringent reconsent trigger would occur before any chimeric integration, where organoids are transplanted into animal models for in vivo testing. This pathway ensures the donor’s autonomy is respected not as a one-time event but as a continuous dialogue, aligning the research process with the evolving nature of the research itself and the core ethical principle of respect for people. The feasibility of this model hinges on robust digital platforms for maintaining secure long-term contact with donors and clearly communicating complex scientific milestones. While resource-intensive, this process ensures ethical rigor and fosters lasting trust between the research community and the public.

### Special provisions: targeted risk mitigation

The Guidelines also impose domain-specific safeguards in the following aspects [1] (Additional file 1: Fig. 1c). For brain organoids, real-time electroencephalogram (EEG) monitoring and complexity caps prevent perithreshold consciousness emergence. However, the reliance on EEG-based monitoring presents significant interpretative challenges. Cerebral organoids generate electrical activity that is often rudimentary, noisy, and prone to false positives. The bursts of activity may be statistically complex but lack the specific spatial–temporal organization correlated with conscious states in intact brains. Over-interpreting these ambiguous signals could lead to the premature termination of valuable research. Therefore, effective implementation of these ‘complexity caps’ requires a hybrid, multi-modal strategy to mitigate technological limitations. This strategy will mandate that EEG activity approaching pre-defined thresholds must be cross-validated against complementary biomarkers before triggering the most stringent regulatory actions. These biomarkers could include: 1) transcriptomic signatures of neuronal maturity and cortical layer specificity; 2) morphological evidence of complex synaptic architecture (e.g., via immunohistochemistry); 3) functional evidence of coordinated, network-wide synchronization (e.g., via calcium imaging). This conservative, multi-parameter approach reduces reliance on any single fallible modality and provides a more robust and scientifically defensible operationalization of the Guidelines’ pioneering ethical safeguards against consciousness risks.

Human-animal chimeras must strictly restrict human cell ratios and need behavioral tracking to avoid species integrity, human germline contamination, and cross-species cognition. For ISEMs, the explicit ban on uterine implantation and culture termination upon neural tube formation can eliminate synthetic embryogenesis completion. These risk-stratified surveillances enforced by electrophysiological thresholds, cell ratio caps, and morphogenetic checkpoints establish the world’s first regulatory framework for neuroethical and ontological risks of human organoid research, with no equivalent in other major jurisdictions.

## Global governance mosaic and comparative analysis of ethical governance of human brain organoids, organoid-chimeras, and ISEMs

The global regulatory landscape of human brain organoids, organoid chimeras, and embryonic models reveals starkly divergent philosophies, as indicated in Additional file 1: Fig. 1d. The U.S. exemplifies patchwork pragmatism, mainly relying on decentralized oversight through guidelines of the National Institutes of Health, institutional review board reviews, and disparate state laws [8, 10]. While prioritizing risk–benefit analysis and imposing federal funding bans on human-animal chimeras, its commercial sector faces minimal constraints, indicating uncertainty in brain organoid and chimera research. In contrast, the European Union (EU) enshrines human dignity as a doctrine, unifying governance under the General Data Protection Regulation, Clinical Trials Regulation, and the Oviedo Convention [8–10]. The EU’s framework imposes non-negotiable bans on human germline editing and extreme caution toward neural organoids, though critics warn its precautionary stance may hinder translational progress. Meanwhile, Australia adopts tiered licensing via its updated National Statement (2023) from the National Health and Medical Research Council (NHMRC), mandating dual approvals for embryo-derived organoids and explicit chimera restrictions while pioneering “ethical phase-gating” for progressive oversight. China’s Guidelines stand out through: 1) systematic centralization via the implementation of unified national standards; 2) tiered risk governance (including foundational principles, general requirements, and explicit special requirements for brain organoids, chimeras, and embryo models, directly confronting ethical “gray zones”); 3) equity emphasis (“fairness and justice” as a core principle, addressing resource allocation and data-sharing ethics); 4) the world’s first national guidelines to codify binding standards for collectively targeting brain organoids, organoid-chimeras, and embryo models (e.g., ISEMs); 5) an absolute ban on ISEM implantation into the uterus of a human or animal host and establishing developmental termination mechanisms (based on the shortest time required to achieve scientific goals) stricter than the “14 d rule”; 6) a preventive governance model that preemptively resolves ethical risks through pre-research assessments and researcher-binding commitments. China’s framework marks its transition from following international norms to defining ethical standards for high-risk emerging biotechnologies, particularly leading global governance in consciousness potential and synthetic embryo oversight.

However, China’s recent guideline belong to a framework rather than detailed technical specifications. More quantitative compliance thresholds should be included to enhance regulatory operability. For cerebral organoid research, the Guidelines only require “strengthening ethical review” and “dynamic monitoring of consciousness risks” [1]. Therefore, tiered oversight scaling with morphological evidence of complex synaptic architecture and high-content microelectrode arrays-based electrophysiological activity (e.g., EEG monitoring) should be clearly integrated into the current Guidelines to trigger intensified ethics review when gamma oscillation intensity exceeds risk thresholds [e.g., sustained (e.g., > 3 s), high-power, spatially synchronized oscillations in high-gamma range (60 − 100 Hz)]. For organoid-chimera research, the Guidelines require “strict control of human cell proportion” and “prevention of germline transmission”, but no upper limit of proportion is set [1]. Stage-dependent termination limits (e.g., capping human cells at ≤ 5% in embryos and ≤ 0.1% postnatally) should be introduced to prevent developmental overreach in chimeras. For ISEMs, the Guidelines require “restriction of development stage” and “prohibition of transplantation into the uterus”, but no termination trigger point is defined [1]. Artificial intelligence (AI)-powered morphological surveillance triggering automatic termination (e.g., 24-hour destruction post-neural tube/primitive streak detection) should be included in the near future to enforce ethical boundaries. Such proposed technical indicators may transform ongoing Guidelines into dynamic, risk-calibrated safeguards that balance innovation with ethical vigilance of human organoids.

## The potential domestic and international impacts of China’s Guidelines

Although comprehensive case studies are pending, early institutional adoption demonstrates the Guidelines’ tangible impact. Preliminary feedback from ethicists and scientists indicates a rapid shift in domestic institutional policies. Several leading universities and institutions in China have publicly revised internal ethical review protocols to align with the recently released national framework. For example, new grant proposals now require detailed appendices proving compliance with specific “special requirements” for high-risk categories like chimeras and brain organoids. This top-down integration of the national framework into institutional and grant-making workflows provides strong preliminary evidence of the Guidelines’ potent influence, effectively translating policy into practice and actively shaping real-time ethical conduct in labs across China.

Beyond its domestic implications, China’s framework presents a significant case study for international harmonization, particularly as the ISSCR prepares its 2026 guidelines. Its influence will likely be felt not through direct replication but by providing a tested model of anticipatory, state-led governance that challenges more decentralized approaches. For instance, its pioneering mandate for real-time electrophysiological monitoring to preempt consciousness emergence in brain organoids directly forces a global conversation on technical standards and ethical thresholds that have thus far been theoretical. Furthermore, the Guidelines’ operationalization of communitarian bioethics through requirements like dynamic consent protocols and strict national management of genetic resources, offers a concrete alternative to individual-centric models, pushing international bodies to consider how principles like collective welfare and equity can be practically implemented. Finally, its three-tiered scrutiny system provides a unified regulatory architecture for the entire field, a feature of interest for policymakers seeking clarity amidst rapid innovation. As a first-mover in comprehensive national regulation, China’s regulatory experiment provides indispensable empirical ground for the ISSCR’s forthcoming deliberations, ensuring they are informed by a wider range of governance experiments beyond Western paradigms. While not all provisions may be directly transferable, China’s proactive stance is poised to stimulate crucial debate and provide a comparative reference point, guaranteeing the ISSCR’s 2026 revisions incorporate a wider range of governance experiments. The engagement of Chinese scientists and ethicists in international forums will be crucial to this process of cross-pollination.

## The implementation challenge of dual compliance: possible pathways for ethical alignment in international collaboration

The Guidelines’ stipulation that international collaboration must satisfy all involved jurisdictions presents a significant implementation challenge, particularly when ethical paradigms clash. A key tension arises between China’s communitarian prioritization of collective welfare and the Western emphasis on individual autonomy, especially concerning informed consent and data sovereignty. To resolve such ethical conflicts in cross-border research, mechanisms beyond a simple dual-compliance model are needed. Potential pathways include the development of bilateral Mutual Recognition Agreements, where accredited ethics committees in partner countries agree to recognize each other’s approvals for specific research types, contingent on adhering to a core set of agreed-upon principles. For more complex projects, the establishment of joint ethical review committees comprising members from both sides could provide a dedicated forum to negotiate compromises on a case-by-case basis. Furthermore, a principle of jurisdictional primacy could be applied, where the location of the biological sample origin dictates the consent process, while the physical location of the experiment governs oversight and monitoring protocols. These mechanisms would transform a potential regulatory deadlock into a structured process for ethical negotiation, ensuring foundational cultural values are respected while enabling crucial global scientific cooperation on organoid research.

## Concluding remarks

The regulatory science paradox always exists. Overly granular standards risk stifling innovation, while excessively fragmented frameworks invite unchecked risks. China navigates a third path by using principles to set boundaries and leveraging technology to refine details. China’s Guidelines hold advantages in positioning clarity and technical ambiguity. The Guidelines mark a global first by establishing parallel special requirements for 3 high-risk research categories, including brain organoids, organoid chimeras, and ISEMs. The next critical step is to transform abstract concepts into executable laboratory standard operating procedures (SOPs). So far, China still requires concrete, technically specific standards within its overarching regulatory framework to further upgrade the current Guidelines and enhance their implementation.

In short, China’s framework demonstrates 3 key advances in bioethical governance, including anticipatory regulation, tiered scrutiny, and cross-disciplinary integration that may serve as a vital reference point for global policymaking. While enforcement mechanisms require strengthening, this model offers a template for international policymakers grappling with human organoid ethics. Its future impacts on global standard-setting hinge on active participation in forums like the ISSCR’s 2026 update process, fostering the international calibration of neuroethical and ontological thresholds and shared investment in monitoring technologies.

## Supplementary Information

**Additional file 1. Fig. S1** The foundational principles, general requirements, special provisions, and comparative analysis of the Guidelines.

## Data Availability

Not applicable.

## Abbreviations

NAM: New Alternative Methodologies
ISEMs: Integrated stem cell-based embryo models
ISSCR: International Society for Stem Cell Research
RECs: Research Ethics Committees
iPSCs: Induced pluripotent stem cells
EEG: Electroencephalogram
EU: European Union
NHMRC: National Health and Medical Research Council
AI: Artificial intelligence
SOPs: Standard operating procedures
