# Supplementary material for: Charting bioethical frontiers: China’s human organoid guidelines in a global context
Source: Mil Med Res. 2025 Oct 9;12:64. doi: 10.1186/s40779-025-00651-x (PMC12509384; doi:10.1186/s40779-025-00651-x)
Supplement: Supplementary file 1 — Additional file 1. Fig. S1 The foundational principles, general requirements, special provisions, and comparative analysis of the Guidelines. [file 40779_2025_651_MOESM1_ESM.pdf]

**a** The 5 foundational principles

| Principles | Ethical foundations | Operational requirements |
|---|---|---|
| Beneficence | Social utilitarianism | Prioritize public health outcomes |
| Risk control | Biosecurity imperatives | Environmental/donor harm mitigation |
| Respect for autonomy | Informed consent tradition | Dynamic re-consent mechanisms |
| Scientific necessity | Resource efficiency norms | Minimal cell use justification |
| Fairness | Equity in healthcare access | Anti-stigmatization protocols |

**b** The 8 general requirements

| Requirements | Contents | Requirements | Contents |
|---|---|---|---|
| Scientific value | Research must advance knowledge with societal benefit | Personnel & facilities | Trained researchers and certified labs |
| Legal compliance | Strict adherence to national/ international laws and ethics codes | Resource management | Traceable handling of biomaterials and genetic resources |
| Ethics review | Mandatory institutional committees with domain expertise | Data governance | Secure data processing aligned with donor agreements |
| Informed consent | Dynamic donor consent with privacy protection | International cooperation | Cross-border projects must satisfy all jurisdictions' regulations |

**c** The 3 special provisions

| Research types | Key restrictions | Scientific rationale |
|---|---|---|
| Brain organoids | Electrophysiological activity monitoring; Complexity caps | Prevent perithreshold consciousness emergence |
| Human-animal chimeras | Strict control of human cell ratio | Limit CNS contribution to host cognition; Risks of cross-species biohybrids |
| ISEMs | Uterine implantation ban; shortest time rule; define termination conditions | Avoid synthetic embryogenesis completion |

**d** The comparative analysis of ethical governance of human brain organoids, human-animal chimeras, and ISEMs

| Subitems | Country/Region | Regulatory specificity | Key technical standards |
|---|---|---|---|
| **Brain organoids** - quantitative differences in consciousness risk regulation | China | Framework-based★★☆ | Dynamic consciousness monitoring (no quantitative indicators) |
| | USA | Loose★☆☆ | NIH bans funding for primate chimeras with human neural cells; No mandatory private-sector monitoring |
| | EU | Conservative★★☆ | 14-day rule default (ban on ≥14-day culture); Germany bans all neural behavioral tests |
| | Japan | Tiered★★☆ | National approval required; Allows low-complexity models (no sensory input/output) |
| | South Korea | Specific★★★ | Brain Organoid Guidelines (2024): Mandatory EEG monitoring; ≥60 Hz γ oscillation density triggers ethics |

| Subitems | Country/Region | Human cell ratio limit | Key constraints |
|---|---|---|---|
| **Organoid-chimeras** - hard limits on human cell ratios | China | Strict control(unquantified) | Bans germline transmission |
| | Japan | ≤5% (embryonic stage) | Bans human cells in gonad/brain development |
| | Australia | ≤1% (adult animals) | Dual licensing: Proof of scientific value+irreplaceability |
| | USA | No federal standard | States (e.g., California) ban human-primate chimeras; NIH projects require ≤0.1% |
| | EU | Banned | Oviedo Convention prohibits all human-animal chimeric embryos |

| Subitems | Country/Region | Development termination point | Technical trigger conditions |
|---|---|---|---|
| **ISEMs** - operational differences in termination mechanisms | China | Limit developmental stages | No defined morphological markers |
| | UK | Primitive streak appearance (approximately 14 d) | Mandatory destruction of primitive axis structures |
| | Australia | Early neural tube (approximately 21 d) | Morphological monitoring+auto-degrading culture media |
| | USA | No unified standard | ISSCR recommends ≤14 d; Private labs often extend to 28 d |
| | International consensus | Gastrulation | Bans body axis establishment and spatial cell fate differentiation |

**Fig. S1** The foundational principles, general requirements, special provisions, and comparative analysis of the Guidelines. CNS central nervous system, ISEMs integrated stem cell-based embryo

models, EEG electroencephalogram, NIH National Institutes of Health, ISSCR International Society for Stem Cell Research
